# Trusted Assessments: Avoiding Duplication of Work, Improving Efficiency and Trusting Colleagues

**DOI:** 10.1192/bjo.2025.10397

**Published:** 2025-06-20

**Authors:** Hannah Pasha Memon, Joanne Hew, Stephen O’Connor, Neha Singh

**Affiliations:** NELFT, London, United Kingdom

## Abstract

**Aims:** Psychiatric patients attending acute hospitals settings in North East London are reviewed by the Psychiatric Liaison Service (PLS). Those who PLS deem to require Older Adults Home Treatment Team (OAHTT) input on discharge are re-assessed face to face by a member of the OAHTT prior to discharge from the acute hospital. This is time consuming as it requires OAHTT staff to travel to the acute hospital and re-assess the patient. This can delay discharge and the outcome of the assessment is rarely different to the decision PLS staff would have made.

The aim of our quality improvement project is to streamline the process of referrals to OAHTT and prevent duplication of work. Referrals made to the OAHTT from PLS at Queen’s Hospital, Romford (QH) and King George Hospital, Ilford (KGH) would be discussed on the phone and accepted for OAHTT follow up or admission without the need to conduct a separate assessment.

**Methods:** Baseline data were collected for all patients referred to the OA HTT in January 2023–Dec 2023 from QH PLS and in July 2023–June 2024 from KGH PLS. The percentage of patients who had face to face assessments by OAHTT were recorded. The trusted assessments intervention was launched in QH in January 2024, and in KGH in July 2024. Following intervention, the percentage of patients who had face to face assessments by the OAHTT were recorded.

**Results:** Prior to intervention, 95% of all referrals made from QH PLS to OA HTT were assessed face to face. This reduced to 25% post-intervention (data from January 2024–November 2024). Therefore, a 75% reduction in face to face assessments was achieved.

In KGH, prior to intervention, 84% of referrals were assessed face to face. Preliminary data (July 2024–November 2024) show that post intervention in KGH, 50% of referrals were assessed face to face. This is a reduction of 40%.

**Conclusion:** The trusted assessment model resulted in a large reduction in face to face assessments conducted by the OAHTT following referral by the PLS teams. This model appears to have achieved its aims of streamlining referrals, preventing duplication of work and improving efficiency. The next step would be to extend this model to adult HTT services and evaluate if the same benefits can be achieved.

